# Supplementary material for: Co-crystal structures of the fluorogenic aptamer Beetroot show that close homology may not predict similar RNA architecture
Source: Nat Commun. 2023 May 23;14:2969. doi: 10.1038/s41467-023-38683-3 (PMC10205801; doi:10.1038/s41467-023-38683-3)
Supplement: Supplementary file 1 — Supplementary Information [file 41467_2023_38683_MOESM1_ESM.pdf]

Co-crystal structures of the fluorogenic aptamer Beetroot show that close homology may not predict similar RNA architecture

Luiz F.M. Passalacqua[1], Mary R. Starich[1], Katie A. Link[1], Jiahui Wu[2,3], Jay R. Knutson[1], Nico Tjandra[1], Samie R. Jaffrey[2] & Adrian R. Ferré-D'Amaré[1*]

[1]Biochemistry and Biophysics Center, National Heart, Lung, and Blood Institute, National Institutes of Health, Bethesda, MD, USA

[2] Department of Pharmacology, Weill-Cornell Medical College, Cornell University, New York, New York, USA

[3] Current address: Department of Chemistry, Binghamton University, Binghamton, NY 13902, USA

*Address correspondence to A.R.F.; e-mail adrian.ferre@nih.gov

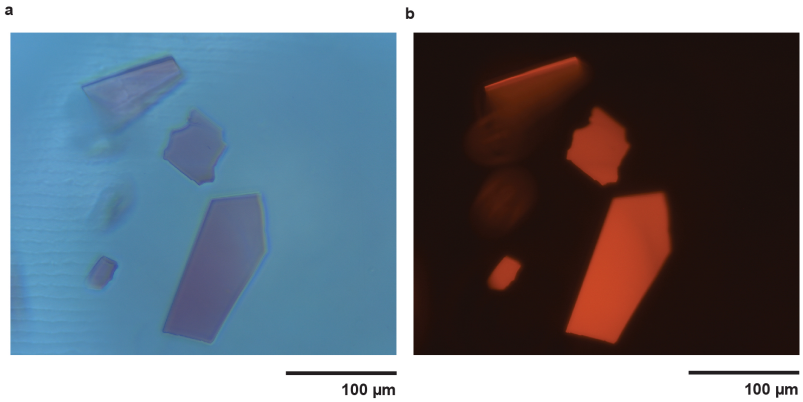

Supplementary Figure 1. Beetroot–DFAME crystal. a, Bright-field photomicrograph of plate-shaped Beetroot–DFAME co-crystals (**Supplementary Table 1)**. **b**, Fluorescence of same crystals under 500 nm illumination. Three independent experiments provided the same result.

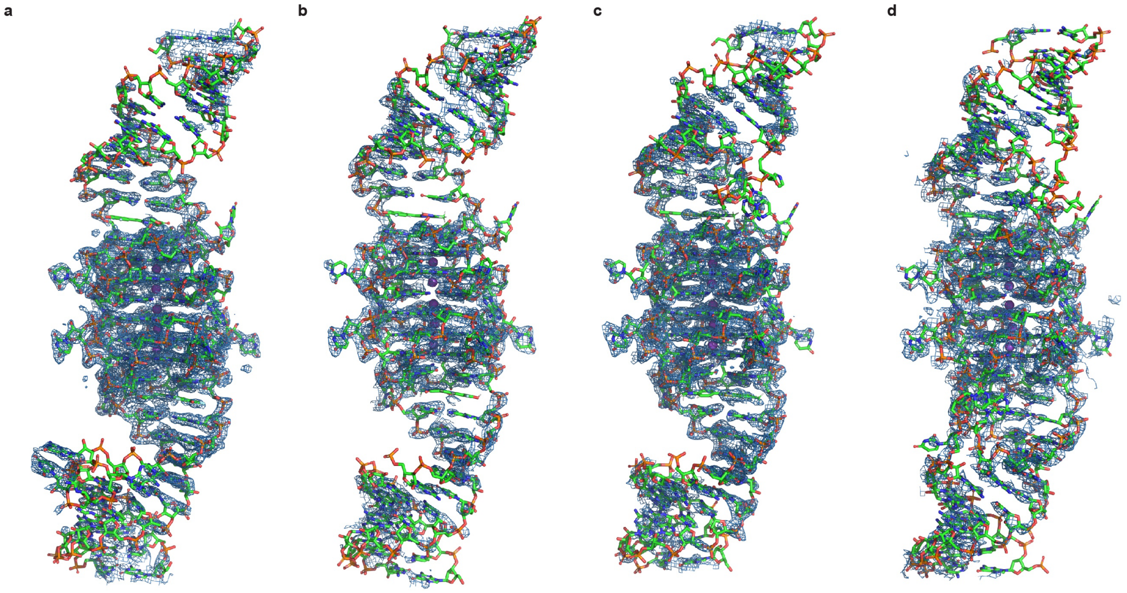

Supplementary Figure 2. Electron density maps for structures determined in this study. Composite simulated annealing-omit 2|F_o| - |F_c | electron density maps for the (**a**) Beetroot–DFAME, (**b**) Beetroot–DFHO, (**c**) Beetroot–ThT, and (**d**) Wobble Beetroot–DFHO complexes contoured at 1.5 σ (navy meshes) for (**a**), (**b**), and (**c**) and at 1.0 σ for (**d**), superimposed on the respective final refined models.

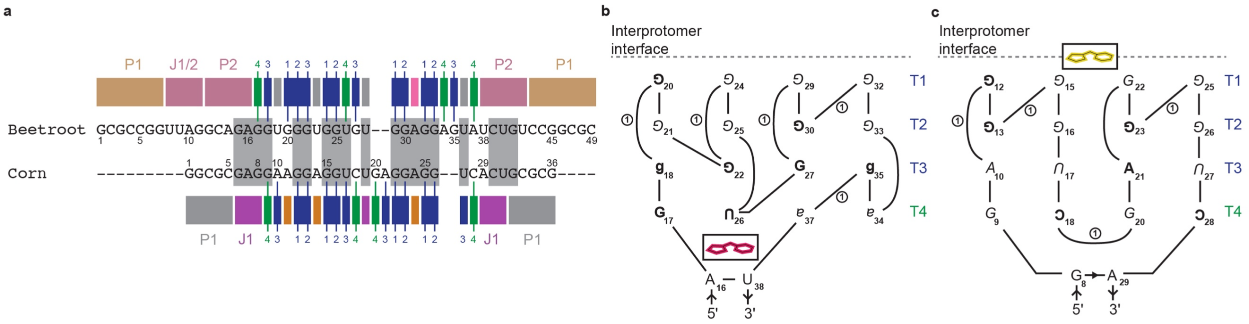

Supplementary Figure 3. Beetroot and Corn sequence alignment and G-quartet comparison. **a,** Sequence alignment of the aptamers Beetroot and Corn, numbered according to the total length of Beetroot and Corn constructs employed in their respective crystallographic structure determinations (this work, and ref. 1, respectively). Conserved nucleotides are boxed in gray and colored boxes indicate structural motifs color coded as in **Fig. 1 c**, **d**, and **e**. Colored lines and numbers indicate nucleotide composition of each quartet tier. **b**, Graphical G-quartet schematics[2] for Beetroot and **c**, Corn, numbered according to Beetroot and Corn registers, respectively. Each row represents the nucleotides of a quartet tier, and columns indicate nucleotide stacks. Upper-case, lower-case, bold, and Italic letters denote *anti*, *syn,* 2′-endo and 3′-endo nucleotides, respectively; upside-down letters denote strand polarity inversion with respect to the 5′-most nucleotide in the scheme. Lines connecting nucleotides are loops and bulges, with the number of nucleotides indicated within a circle.

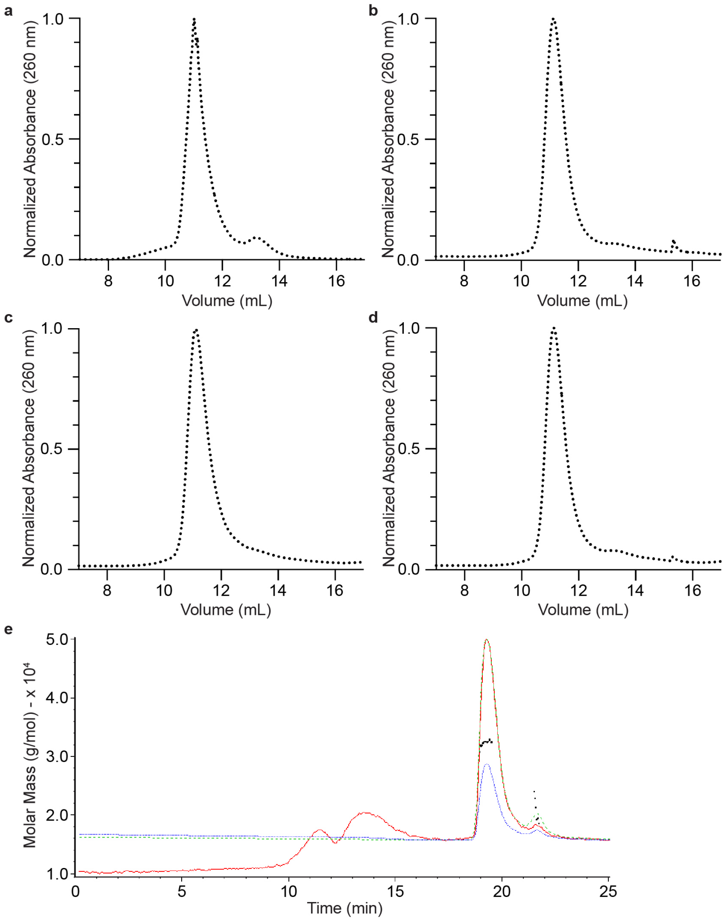

Supplementary Figure 4. Size-exclusion chromatographic (SEC) analysis of unliganded Beetroot. a, SEC of unliganded, freshly folded Beetroot (**Methods**), (**b**) one hour after folding, (**c**) four hours after folding, and (**d**) 24 hours after folding. **e**, SEC-multi-angle light scattering (SEC-MALS) analysis of unliganded Beetroot. Dashed green line is absorbance at 280 nm, blue line is the refractive index, red line is light scattering, and black dots correspond to calculated molar mass.

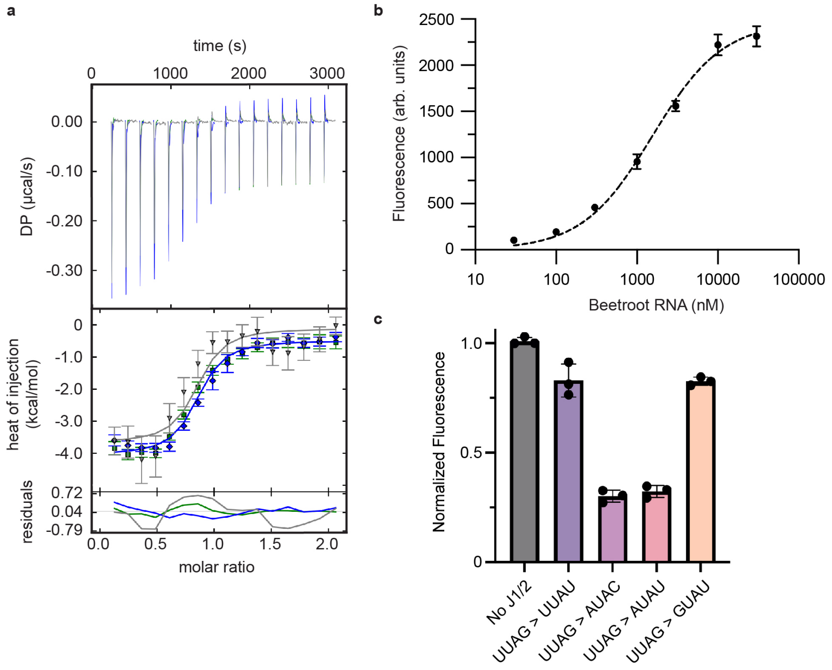

Supplementary Figure 5. Characterization of the Beetroot–DFAME complex. a, Isothermal titration calorimetry triplicate (including data from **Fig. 2g**), baseline-corrected thermograms, fits, and residuals for Beetroot titrated with DFAME (means ± s.d., n = 3 – n means independent experiments). ΔH, ΔG, and $T\Delta S$ are -3.58 ± 0.13 kcal/Mol, -8.67 ± 0.25 kcal/Mol, and 5.08 ± 0.37 kcal/Mol, respectively. Calculated K_d is 0.47 ± 0.18 µM. **b**, Fluorescence of DFAME titrated with Beetroot RNA (mean ± s.d., n = 3 – n means independent sample replicate). Calculated K_d is 1.55 ± 0.23 µM. **c**, Fluorescence of DFAME in the presence of Beetroot J1/2 region mutants, normalized to the fluorescence with the wild-type (mean ± s.d., n = 3 – n means independent sample replicate).

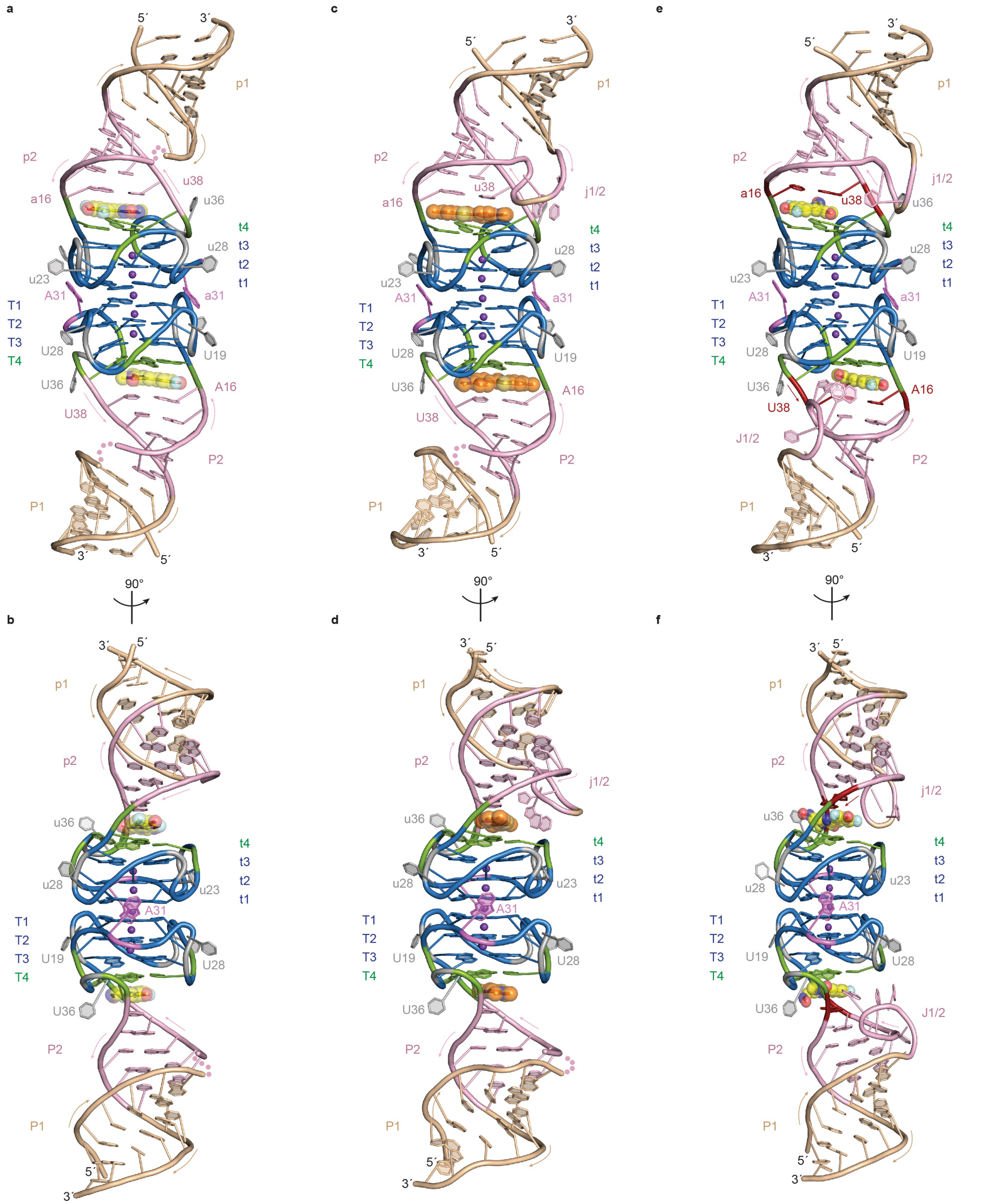

Supplementary Figure 6. Structures of Beetroot–DFHO, Beetroot–ThT, and Wobble Beetroot–DFHO complexes. a, Cartoon representation of the Beetroot–DFHO dimeric complex.

Arrows indicate 5′ to 3′ chain direction, and purple spheres represent K^+. The bound DFHO molecules are shown in ball-and-stick representation with translucent spheres. **b**, Orthogonal view of **a**. **c**, Same as (**a**) for the Beetroot–ThT complex. **d**, Orthogonal view of **c**. **e**, Same as (**a**) for the Wobble Beetroot–DFHO complex. Mutated nucleotides are in red. **f**, Orthogonal view of **e**.

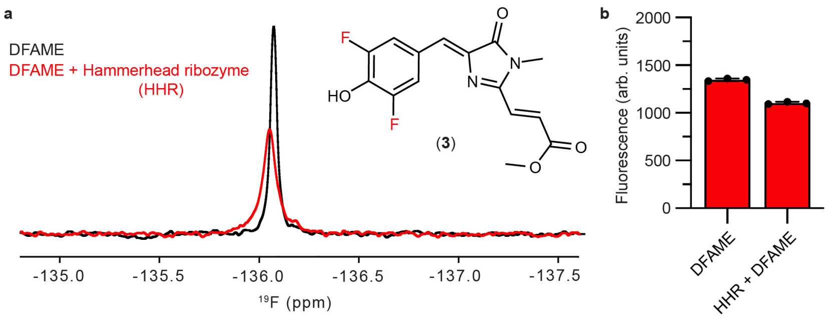

Supplementary Figure 7. Fluorophore dynamics of DFAME in the presence of hammerhead ribozyme. a, ^{19}F NMR spectra of DFAME free and in the presence of hammerhead ribozyme (HHR). Insets indicate the fluorines of the fluorophores assigned to the observed resonances. **b**, Background fluorescence of DFAME and fluorescence activation of DFAME in the presence of HHR (mean ± s.d., n = 3 – n means independent sample replicate).

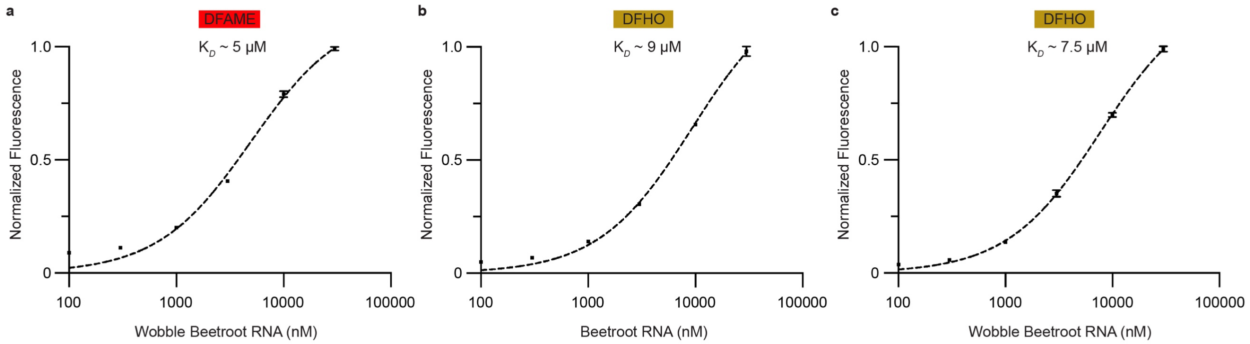

Supplementary Figure 8. Dissociation constants of Beetroot and Wobble Beetroot. a, Fluorescence of DFAME titrated with Wobble Beetroot RNA. Calculated K_d is 4.9 ± 1.2 µM. **b**, Fluorescence of DFHO titrated with Beetroot RNA. Calculated K_d is 9.3 ± 1.5 µM **c**, Fluorescence of DFHO titrated with Wobble Beetroot RNA. Calculated K_d is 7.6 ± 0.8 µM. (mean ± s.d., n = 3 – n means independent sample replicate)

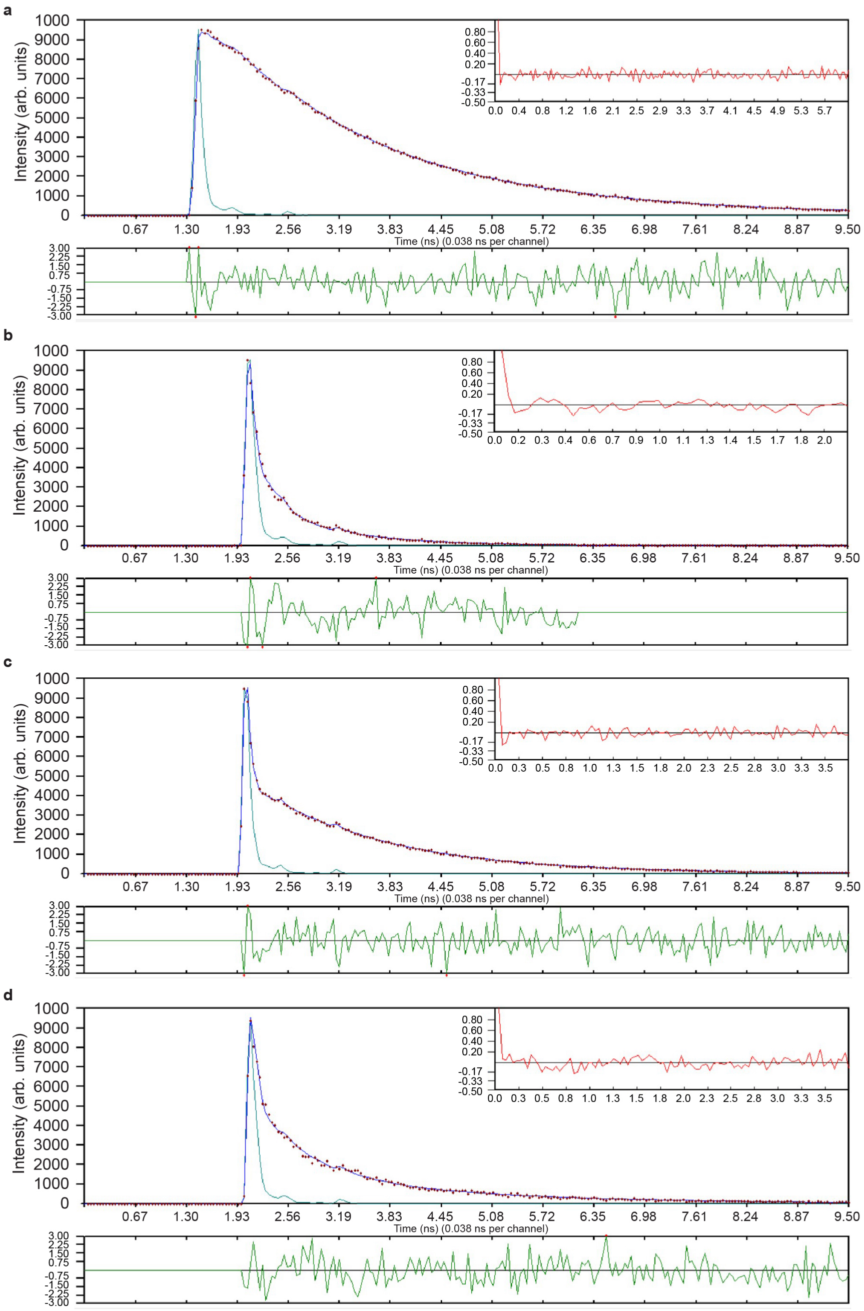

Supplementary Figure 9. Fluorescence lifetimes of fluorophore–Beetroot complexes. Fluorescence lifetime traces of (**a**) Beetroot–DFAME, (**b**) Beetroot–DFHO, (**c**) Wobble Beetroot–DFAME, (**d**) Wobble Beetroot–DFHO. The fluorescence lifetime decay (red points) with fit (dark blue) and instrumental function (light blue). Below in green is the weighted residuals and in the top right is the autocorrelation (red).

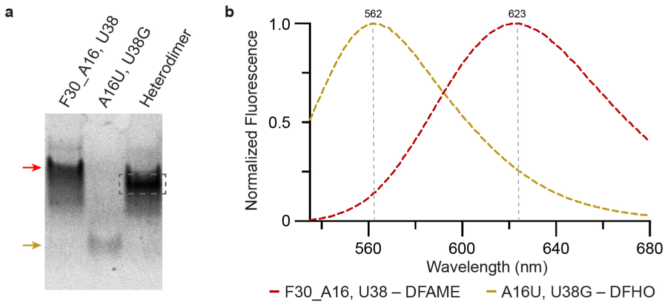

Supplementary Figure 10. Stability of the heterodimer and spectra of homodimeric F30_wild-type Beetroot and Wobble Beetroot. **a**, Resolved native polyacrylamide gel electrophoresis of the heterodimer 48 hours after purification of the complex. Dashed box indicate heterodimer band, red arrow indicates F30 fused wild-type homodimer control, and mustard arrow indicates Wobble homodimer control. Two independent experiments provided the same result. **b**, Emission spectra of homodimeric F30_wild-type Beetroot bound to DFAME and homodimeric Wobble Beetroot bound to DFHO. Both samples were excited at 470 nm.

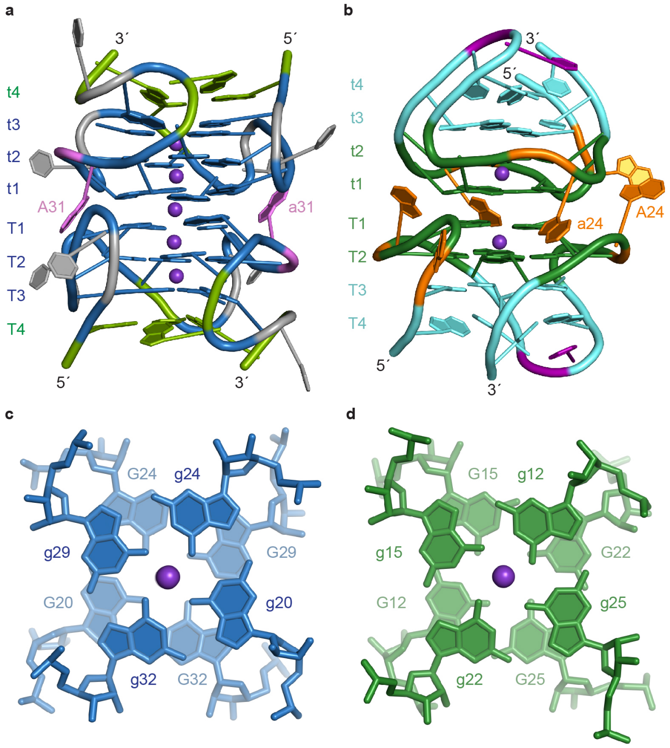

Supplementary Figure 11. Comparison of Beetroot and unliganded Corn interfaces. a, Side view of the eight-tiered quadruplex of Beetroot homodimer. **b**, Side view of the eight-tiered quadruplex of unliganded Corn homodimer[3] (PDB: 6e80). **c**, Top-down view of the interprotomer interface of Beetroot with the top G-quartet tier (t1 and T1) of each protomer and K^+ ions. **d**, Top-down view of the interprotomer interface of unliganded Corn top G-quartet tier (t1 and T1) of each protomer and K^+ ions. Images were aligned to the first 5'-guanosine of T1 of each molecule (G20 of Beetroot and G12 of Corn).

Supplementary Table 1. Crystallographic statistics

	Beetroot–DFAME	Beetroot–DFHO	Beetroot–ThT	Beetroot A16U-U38G–DFHO
**Data collection*†**				
Beamline	ALS 5.02	APS 24-ID-C	APS 23-ID-D	ALS 5.02
Wavelength (Å)	0.977	1.104	1.033	1.000
Space group	$P\ 12_11$	$P\ 12_11$	$P\ 12_11$	$P\ 12_11$
Cell dimensions				
a, *b*, *c* (Å)	29.94 50.74 95.55	29.79 50.79 97.09	29.69 50.57 94.74	30.01 50.35 94.58
α, β, γ (°)	90 94.08 90	90 94.90 90	90 93.18 90	90 95.70 90
Resolution (Å)	47.66 - 1.95	96.73 - 2.55	47.3 - 2.1	94.12 - 2.85
R_{merge}	0.0557 (0.236)	0.139 (0.3438)	0.2148 (1.133)	0.2947 (1.186)
$CC_{1/2}$	0.999 (0.985)	0.994 (0.966)	0.992 (0.893)	0.989 (0.482)
$<I>/<\sigma(I)>$	16.32 (2.78)	8.08 (1.46)	7.16 (1.84)	2.63 (0.45)
Completeness (%)	99.82 (99.67)	98.72 (98.07)	99.55 (98.70)	99.88 (90.47)
Redundancy	6.5 (6.7)	6.5 (5.9)	5.6 (5.7)	6.4 (5.9)
**Refinement**				
Resolution (Å)	47.66 - 1.95 (2.02 - 1.95)	48.37 - 2.55 (2.64 - 2.55)	47.3 - 2.1 (2.17 - 2.1)	47.06 - 2.85 (2.95 - 2.85)
No. reflections	20999 (2088)	9499 (915)	16467 (1599)	6619 (617)
R_{work} / R_{free}	0.179 / 0.204	0.183 / 0.210	0.180 / 0.220	0.194 / 0.256
No. atoms				
RNA	2027	1952	2063	2073
Ligand/ion	51	45	45	45
Water	148	49	113	35
B-factors (Å^2)				
RNA	66.07	83.45	67.66	60.78
Ligand/ion	42.00	66.03	34.25	28.25
Water	37.30	36.79	40.49	23.00
R.m.s. deviations				
Bond lengths (Å)	0.004	0.006	0.006	0.007
Bond angles (°)	0.82	1.20	1.15	1.15
Mean precision (Å)	0.18	0.31	0.23	0.43
PDB ID	8EYU	8EYV	8EYW	8F0N

*One crystal was used for each dataset. †Values in parentheses are for highest resolution shell.

Supplementary Table 2. Sequences of RNAs used in this study.

Name	RNA Sequence, 5' to 3'
Beetroot	GCGCCGGUUAGGCAGAGGUGGGUGGUGUGGAGGAGUAUCUGUCCGGCGC
A31C	GCGCCGGUUAGGCAGAGGUGGGUGGUGUGGCGGAGUAUCUGUCCGGCGC
A31U	GCGCCGGUUAGGCAGAGGUGGGUGGUGUGGUGGAGUAUCUGUCCGGCGC
A16G	GCGCCGGUUAGGCAGGGGUGGGUGGUGUGGAGGAGUAUCUGUCCGGCGC
A16C	GCGCCGGUUAGGCAGCGGUGGGUGGUGUGGAGGAGUAUCUGUCCGGCGC
A16U	GCGCCGGUUAGGCAGUGGUGGGUGGUGUGGAGGAGUAUCUGUCCGGCGC
U38G	GCGCCGGUUAGGCAGAGGUGGGUGGUGUGGAGGAGUAGCUGUCCGGCGC
U38C	GCGCCGGUUAGGCAGAGGUGGGUGGUGUGGAGGAGUACCUGUCCGGCGC
A16G + U38C	GCGCCGGUUAGGCAGGGGUGGGUGGUGUGGAGGAGUACCUGUCCGGCGC
A16C + U38G	GCGCCGGUUAGGCAGCGGUGGGUGGUGUGGAGGAGUAGCUGUCCGGCGC
A16U + U38G (Wobble Beetroot)	GCGCCGGUUAGGCAGUGGUGGGUGGUGUGGAGGAGUAGCUGUCCGGCGC
A16U + U38A	GCGCCGGUUAGGCAGUGGUGGGUGGUGUGGAGGAGUAACUGUCCGGCGC
A16U + U38I *	GCGCCGGUUAGGCAGUGGUGGGUGGUGUGGAGGAGUAICUGUCCGGCGC
J1/2 deletion	GCGCCG_GGCAGAGGUGGGUGGUGUGGAGGAGUAUCUGUCCGGCGC
J1/2 UUAG>UUAU	GCGCCGGUUAUGCAGAGGUGGGUGGUGUGGAGGAGUAUCUGUCCGGCGC
J1/2 UUAG>AUAC	GCGCCGGAUACGCAGAGGUGGGUGGUGUGGAGGAGUAUCUGUCCGGCGC
J1/2 UUAG>AUAU	GCGCCGGAUAUGCAGAGGUGGGUGGUGUGGAGGAGUAUCUGUCCGGCGC
J1/2 UUAG>GUAU	GCGCCGGGUAUGCAGAGGUGGGUGGUGUGGAGGAGUAUCUGUCCGGCGC
U26G + A34G**	GCGCCGGUUAGGCAGAGGUGGGUGGGGUGGAGGGGUAUCUGUCCGGCGC
G20A + G24A**	GCGCCGGUUAGGCAGAGGUAGGUAGUGUGGAGGAGUAUCUGUCCGGCGC
G20U**	GCGCCGGUUAGGCAGAGGUUGGUGGUGGGGAGGAGUAUCUGUCCGGCGC
G20U + G29U**	GCGCCGGUUAGGCAGAGGUUGGUGGUGUUGAGGAGUAUCUGUCCGGCGC
G29U**	GCGCCGGUUAGGCAGAGGUGGGUGGUGUUGAGGAGUAUCUGUCCGGCGC
G20U + G32U**	GCGCCGGUUAGGCAGAGGUUGGUGGUGUGGAUGAGUAUCUGUCCGGCGC
G20del + G24del + G29del + G32del**	GCGCCGGUUAGGCAGAGGU_GGU_GUGU_GA_GAGUAUCUGUCCGGCGC
U19del + G20del + G24del + G29del + G32del**	GCGCCGGUUAGGCAGAGG__GGU_GUGU_GA_GAGUAUCUGUCCGGCGC
U19del + G20del + U23del + G24del + G29del + G32del**	GCGCCGGUUAGGCAGAGGU_GG__GUGU_GA_GAGUAUCUGUCCGGCGC
U19del + G20del + G24del +	GCGCCGGUUAGGCAGAGGU_GGU_GUG__GA_GAGUAUCUGUCCGGCGC

U28del + G29del + G32del**	
F30_Beetroot***	GGAGCCAUGUGUAUGUGGUCC<br>GCGCCGGUUAGGCAGAGGUGGGUGGUGUGGAGGAGUAUCUGUCCGGCGC<br>GGACCACAUACUCUGAUGAUCCUUCGGGAUCAUUCAUGGCUCC
Hammerhead ribozyme_Beetroot ****	GGGAGAGcatguggaaacacaugaa<u>CTAACCGGCGC</u><br>CUGAUGAGUCCGUGAGGACGAAACGGUACCCGGUACCGUC<br><u>GCGCCGGUUAG</u>GCAGAGGUGGGUGGUGUGGAGGAGUAUCUGUCCGGCGC

* Custom synthesized by Dharmacon.

** Mutants that did not yield active molecules (results not shown)

*** F30 sequences are in gray.

**** 5′-hammerhead ribozyme served to generate 5′-OH termini. Ribozyme sequences are in blue. Lower-case sequence added to increase molecular weight of ribozyme for proper separation of cleaved products in denaturing PAGE. Underlined sequence is complementary to first 11 nucleotides of Beetroot.

Supplementary References

1. Warner, K.D. et al. A homodimer interface without base pairs in an RNA mimic of red fluorescent protein. *Nat. Chem. Biol.* **13**, 1195-1201 (2017).
2. Banco, M.T. & Ferré-D'Amaré, A.R. The emerging structural complexity of G-quadruplex RNAs. *RNA* **27**, 390-402 (2021).
3. Sjekloća, L. & Ferré-D'Amaré, A.R. Binding between G quadruplexes at the homodimer interface of the Corn RNA aptamer strongly activates Thioflavin T fluorescence. *Cell Chem. Biol.* **26**, 1159-1168 (2019).
