# Supplementary material for: Co-crystal structures of the fluorogenic aptamer Beetroot show that close homology may not predict similar RNA architecture
Source: Nat Commun. 2023 May 23;14:2969. doi: 10.1038/s41467-023-38683-3 (PMC10205801; doi:10.1038/s41467-023-38683-3)
Supplement: Supplementary file 3 — Reporting Summary [file 41467_2023_38683_MOESM3_ESM.pdf]

nature portfolio

Corresponding author(s): Adrian R. Ferré-D'Amaré

Last updated by author(s): Apr 24, 2023

Reporting Summary

Nature Portfolio wishes to improve the reproducibility of the work that we publish. This form provides structure for consistency and transparency in reporting. For further information on Nature Portfolio policies, see our Editorial Policies and the Editorial Policy Checklist.

Statistics

For all statistical analyses, confirm that the following items are present in the figure legend, table legend, main text, or Methods section.

n/a	Confirmed	
☐	☒	The exact sample size (*n*) for each experimental group/condition, given as a discrete number and unit of measurement
☐	☒	A statement on whether measurements were taken from distinct samples or whether the same sample was measured repeatedly
☐	☒	The statistical test(s) used AND whether they are one- or two-sided<br>*Only common tests should be described solely by name; describe more complex techniques in the Methods section.*
☒	☐	A description of all covariates tested
☒	☐	A description of any assumptions or corrections, such as tests of normality and adjustment for multiple comparisons
☐	☒	A full description of the statistical parameters including central tendency (e.g. means) or other basic estimates (e.g. regression coefficient) AND variation (e.g. standard deviation) or associated estimates of uncertainty (e.g. confidence intervals)
☒	☐	For null hypothesis testing, the test statistic (e.g. *F*, *t*, *r*) with confidence intervals, effect sizes, degrees of freedom and *P* value noted<br>*Give P values as exact values whenever suitable.*
☒	☐	For Bayesian analysis, information on the choice of priors and Markov chain Monte Carlo settings
☒	☐	For hierarchical and complex designs, identification of the appropriate level for tests and full reporting of outcomes
☒	☐	Estimates of effect sizes (e.g. Cohen's *d*, Pearson's *r*), indicating how they were calculated

Our web collection on statistics for biologists contains articles on many of the points above.

Software and code

Policy information about availability of computer code

Data collection	Xia2 0.6.475 (academic, open source), Dials 2.2.10 (academic, open source) , XDS V. Jan 31, 2020 (academic, open source).
Data analysis	Phaser-MR 2.8.3 (academic, open source), Coot 0.9.5 (academic, open source), Phenix 1.19.2 (academic, open source), Pymol 2.5.1 (Schrödinger Inc., academic and commercial), AREAIMOL - CCP4 7.1.015 (academic, open source), DecayFit 1.4 (academic, open source), TopSpin v. 3.0 (Bruker Corp., Commercial), NITPIC 1.3.0 (academic, open source), SEDPHAT 15.2b (academic, open source), ASTRA 7 (WyaTT Technology, commercial).

For manuscripts utilizing custom algorithms or software that are central to the research but not yet described in published literature, software must be made available to editors and reviewers. We strongly encourage code deposition in a community repository (e.g. GitHub). See the Nature Portfolio guidelines for submitting code & software for further information.

Data

Policy information about availability of data

All manuscripts must include a data availability statement. This statement should provide the following information, where applicable:

- Accession codes, unique identifiers, or web links for publicly available datasets
- A description of any restrictions on data availability
- For clinical datasets or third party data, please ensure that the statement adheres to our policy

The atomic coordinates and structure factor amplitudes have been deposited with Protein Data bank under accession codes 8EYU (Beetroot–DFAME) [https://

www.rcsb.org/structure/8EYU], 8EYV (Beetroot–DFHO) [https://www.rcsb.org/structure/8EYV], 8EYW (Beetroot–ThT) [https://www.rcsb.org/structure/8EYW], and 8F0N (Beetroot A16U-U38G–DFHO) [https://www.rcsb.org/structure/8F0N]. The RNA aptamer Corn data used in this study is available in the Protein Data bank database under accession code 5BJO [https://www.rcsb.org/structure/5bjo]. The fluorescence, uncropped gels, pictures of the plate-shaped Beetroot–DFAME co-crystals, size-exclusion chromatography, and isothermal titration calorimetry data generated in this study are provided in the Source Data file. Any additional data required will be made available upon request.

Human research participants

Policy information about studies involving human research participants and Sex and Gender in Research.

Reporting on sex and gender	N/A
Population characteristics	N/A
Recruitment	N/A
Ethics oversight	N/A

Note that full information on the approval of the study protocol must also be provided in the manuscript.

Field-specific reporting

Please select the one below that is the best fit for your research. If you are not sure, read the appropriate sections before making your selection.

☒ Life sciences ☐ Behavioural & social sciences ☐ Ecological, evolutionary & environmental sciences

For a reference copy of the document with all sections, see nature.com/documents/nr-reporting-summary-flat.pdf

Life sciences study design

All studies must disclose on these points even when the disclosure is negative.

Sample size	At least triplicates were used when applicable. The minimal number of replicates were chosen to support statistical validation, such as means ± s.d.
Data exclusions	No data excluded.
Replication	At least triplicates were used when applicable. All replicates were successfull.
Randomization	No randomization needed for the study, as selection biases were not an issue in the in-vitro investigations. No cell, animal, or human experiment that would require such was performed in this study.
Blinding	Blinding to avoid observer bias was not an issue in the in-vitro investigations. No cell, animal, or human experiment that would require blinding was performed in this study.

Reporting for specific materials, systems and methods

We require information from authors about some types of materials, experimental systems and methods used in many studies. Here, indicate whether each material, system or method listed is relevant to your study. If you are not sure if a list item applies to your research, read the appropriate section before selecting a response.

Materials & experimental systems

n/a	Involved in the study	
☒	☐	Antibodies
☒	☐	Eukaryotic cell lines
☒	☐	Palaeontology and archaeology
☒	☐	Animals and other organisms
☒	☐	Clinical data
☒	☐	Dual use research of concern

Methods

n/a	Involved in the study	
☒	☐	ChIP-seq
☒	☐	Flow cytometry
☒	☐	MRI-based neuroimaging
